# Hybrid Plasmonic Fiber-Optic Sensors

**DOI:** 10.3390/s20113266

**Published:** 2020-06-08

**Authors:** Miao Qi, Nancy Meng Ying Zhang, Kaiwei Li, Swee Chuan Tjin, Lei Wei

**Affiliations:** 1 School of Electrical and Electronic Engineering and the Photonics Institute, Nanyang Technological University, 50 Nanyang Avenue, Singapore 639798, Singapore; miao001@e.ntu.edu.sg (M.Q.); mzhang018@e.ntu.edu.sg (N.M.Y.Z.); 2 Institute of Photonics Technology, Jinan University, Guangzhou 510632, China; likaiwei11@163.com

**Keywords:** optical fibers, hybrid plasmonic sensors, surface plasmon resonance, localized surface plasmon resonance, 2D materials, graphene, transition metal oxides, gold nanoparticles, cyclodextrin

## Abstract

With the increasing demand of achieving comprehensive perception in every aspect of life, optical fibers have shown great potential in various applications due to their highly-sensitive, highly-integrated, flexible and real-time sensing capabilities. Among various sensing mechanisms, plasmonics based fiber-optic sensors provide remarkable sensitivity benefiting from their outstanding plasmon–matter interaction. Therefore, surface plasmon resonance (SPR) and localized SPR (LSPR)-based hybrid fiber-optic sensors have captured intensive research attention. Conventionally, SPR- or LSPR-based hybrid fiber-optic sensors rely on the resonant electron oscillations of thin metallic films or metallic nanoparticles functionalized on fiber surfaces. Coupled with the new advances in functional nanomaterials as well as fiber structure design and fabrication in recent years, new solutions continue to emerge to further improve the fiber-optic plasmonic sensors’ performances in terms of sensitivity, specificity and biocompatibility. For instance, 2D materials like graphene can enhance the surface plasmon intensity at the metallic film surface due to the plasmon–matter interaction. Two-dimensional (2D) morphology of transition metal oxides can be doped with abundant free electrons to facilitate intrinsic plasmonics in visible or near-infrared frequencies, realizing exceptional field confinement and high sensitivity detection of analyte molecules. Gold nanoparticles capped with macrocyclic supramolecules show excellent selectivity to target biomolecules and ultralow limits of detection. Moreover, specially designed microstructured optical fibers are able to achieve high birefringence that can suppress the output inaccuracy induced by polarization crosstalk and meanwhile deliver promising sensitivity. This review aims to reveal and explore the frontiers of such hybrid plasmonic fiber-optic platforms in various sensing applications.

## 1. Introduction

Plasmonic fiber-optic sensors have captured intensive research attention in recent years due to their high degree of integration, high sensitivity, flexibility and remote-sensing capability [1,2,3]. Fiber-optic plasmonic sensors can be generally classified into two categories, surface plasmon resonance (SPR)-based sensors and localized surface plasmon resonance (LSPR)-based sensors. Conventionally, SPR- and LSPR-based hybrid fiber-optic sensors are realized by depositing thin metal films and metallic nanoparticles on various fiber structures (e.g., fiber gratings, side-polished fiber, microfiber, etc.) respectively to contribute strong plasmon–matter interaction. To further improve the measurement accuracy, sensitivity and selectivity to analyte molecules, specially designed fiber structures or functional materials are normally applied to strengthen the intensity of surface plasmon or the adsorption to target molecules. Specialty optical fibers like microstructured optical fibers (MOFs) can achieve high-level integration so that the very small dimension waveguide and the microfluidic channels are able to be integrated within a single fiber with only micrometer-scale diameter, leading to effective plasmon–matter interaction. The recent breakthroughs in 2D materials such as graphene, transition metal dichalcogenides (MX_2_), transition metal oxides (TMOs), etc. reveal new opportunities in plasmon–matter enhancement by constructing 2D material/metal hybrid plasmonic structures or heavily doping-free carriers in 2D TMOs to realize intrinsic strong plasmonics in frequently used visible or near-infrared (NIR) optical windows. In addition, macrocyclic supramolecules have been recently proven to be excellent surface functionalization candidates for metallic nanoparticles, contributing to a simple functionalization process, selective target molecules recognition and improved biocompatibility.

In this review, the background and the state-of-the-art of SPR/LSPR fiber-optic sensors will be reviewed. More importantly, the aforementioned emerging hybrid fiber-optic plasmonic sensing solutions will be illustrated in detail. For instance, the exploration of how the highly birefringent MOF based SPR sensor can suppress polarization crosstalk and improve sensitivity in the meantime; how to integrate graphene-on-gold hybrid structure on the fiber-optic platform to strengthen the surface plasmon intensity and to effectively adsorb biomolecules; how to dope abundant free electrons in 2D MoO_3_ and achieve highly integrated microfiber based plasmonic sensing in NIR optical frequencies; how to synthesize cyclodextrin-capped gold nanoparticles (AuNPs) in a one-step process and realize microfiber based highly selective detection of cholesterol in human serum, etc. will be demonstrated.

## 2. Fiber-Optic Surface Plasmon Resonance Sensors

**Figure 1:**
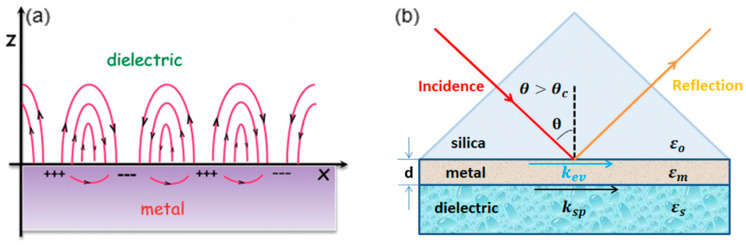
(**a**) The propagating surface plasmon polariton (SPP) at the metal–dielectric interface (Figure adapted with permission from reference [4]). (**b**) The schematic illustration of conventional Kretschmann-Raether prism configuration (Figure adapted with permission from reference [5]).

A surface plasmon polariton (SPP) is an electromagnetic wave that propagates in parallel at the interface between the metal film and dielectric medium. The SPP is TM-polarized. As illustrated in Figure 1a, the polarization direction of SPP is perpendicular to the metal–dielectric interface [4]. The SPP has the evanescent nature, which is strongest at the surface of the metal film and exponentially decays into the dielectric material. Conventionally, SPR is realized on a Kretschmann–Raether silica prism of which the base is coated with a nanometer-scale thin metal film (Figure 1b) [5]. The ambient medium of the thin metal film is dielectric and considered as semi-infinite, by solving the Maxwell equations, the propagation constant of SPP is given by:

(1) $$K_{SP}=\frac{\omega }{c}\sqrt{\frac{\varepsilon_{metal}\varepsilon_{dielectric}}{\varepsilon_{metal}+\varepsilon_{dielectric}}}$$

where *ω*, *c* and *ε* are the frequency of the TM-polarized incident light, light velocity and dielectric constant, independently. The propagation constant of the evanescent wave at the interface is:

(2) $$K_{ev}=\frac{\omega }{c}\sqrt{\varepsilon_{prism}}\sin\theta$$

where *θ* is the angle of light incidence. SPR can be excited by the TM-polarized total reflected light at the silica-metal interface when the phase matches, that the propagation constant of reflected light equals to the propagation constant of SPP:

(3) $$\frac{\omega }{c}\sqrt{\varepsilon_{prism}}\sin\left(\theta_{res}\right)=\frac{\omega }{c}\sqrt{\frac{\varepsilon_{metal}\varepsilon_{dielectric}}{\varepsilon_{metal}+\varepsilon_{dielectric}}+∆\beta }$$

The Δ*β* of the right expression denotes the effects of finite metal layer thickness and high refractive index of prism in real situation. In most fiber-optic SPR sensors, the wavelength interrogation method is employed. The refractive index sensitivity is defined as:

(4) $$S_{n}=\delta \lambda_{res}/\delta n_{s}$$

where *δn_s_* is the change in the refractive index of the analyte and *δλ_res_* is the shift of resonant wavelength [6].

**Figure 2:**
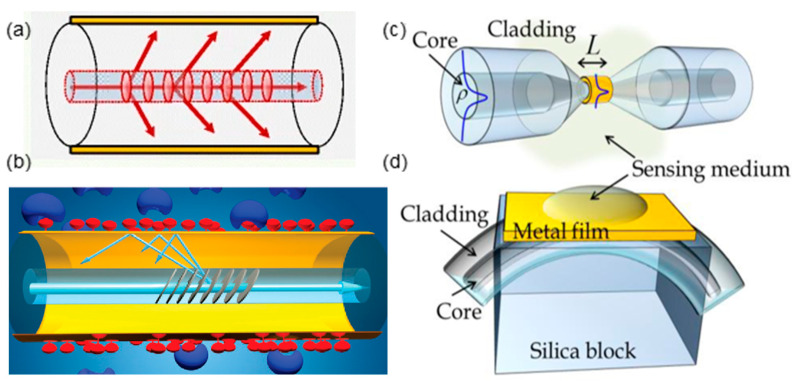
Fiber-optic surface plasmon resonance (SPR) sensor based on (**a**) long-period fiber grating (LPG) (Figure adapted with permission from reference [12]). (**b**) tilted fiber Bragg grating (TFBG) (Figure adapted with permission from reference [15]). (**c**) tapered fiber. (**d**) side-polished fiber (Figure (**c**) and (**d**) adapted with permission from reference [18]).

Along with the increasing demand for compact, highly-integrated, flexible and even in situ sensing devices, optical fiber-based SPR sensors receive more and more attention [7,8,9,10,11]. Various fiber-optic SPR configurations have been investigated to achieve highly sensitive SPR sensors. The key point in the design of the fiber-optic SPR sensor is to realize the phase-matching between the guided mode in fiber and the SPP at the metal-dielectric interface. Hence it is essential to coat the thin metal film at the surface of fiber structure where a strong evanescent field of guide mode can be exposed, leading to the strong SPP at the metal surface for effective light–matter interaction. Fiber gratings like long-period fiber grating (LPG) [12,13,14] and tilted fiber Bragg grating (TFBG) [15,16,17], tapered fiber [18,19,20,21,22,23], side-polished fiber [18,24], etc. have been demonstrated to be feasible for SPR sensing (Figure 2).

In recent years, MOFs are favored due to higher degree of integration, longer interaction distance and improved robustness, that the cladding air holes can function as microfluidic channels for liquid or gas analyte infiltration [25,26,27]. With the distinctive design of core dimension and cladding air holes arrangement, the thin metal films coated on the inner surface of air holes can effectively interact with the evanescent field of the core mode, which grants access to infiltrated analyte to the strong SPP. Numerous MOF structures have been proposed, including hexagonal MOFs [28], semicircular- channel MOFs [29], exposed-core MOFs [30], etc. In most MOF-based SPR designs, the prime consideration is to facilitate simple analyte infiltration and large interaction area. Hence, birefringence commonly exists in MOF-based SPRs. Based on Equation (1), birefringence leads to the offset between SPR wavelengths corresponding to two orthogonal polarizations of core mode. When external perturbations such as fiber bending, twisting and pressure are applied on the fiber, the coupling from the desired mode polarization to undesired mode polarization will occur. Therefore, the overall SPR peak, which is the superposition of SPR of two orthogonal polarizations, will be unstable, leading to inaccurate sensing results.

**Figure 3:**
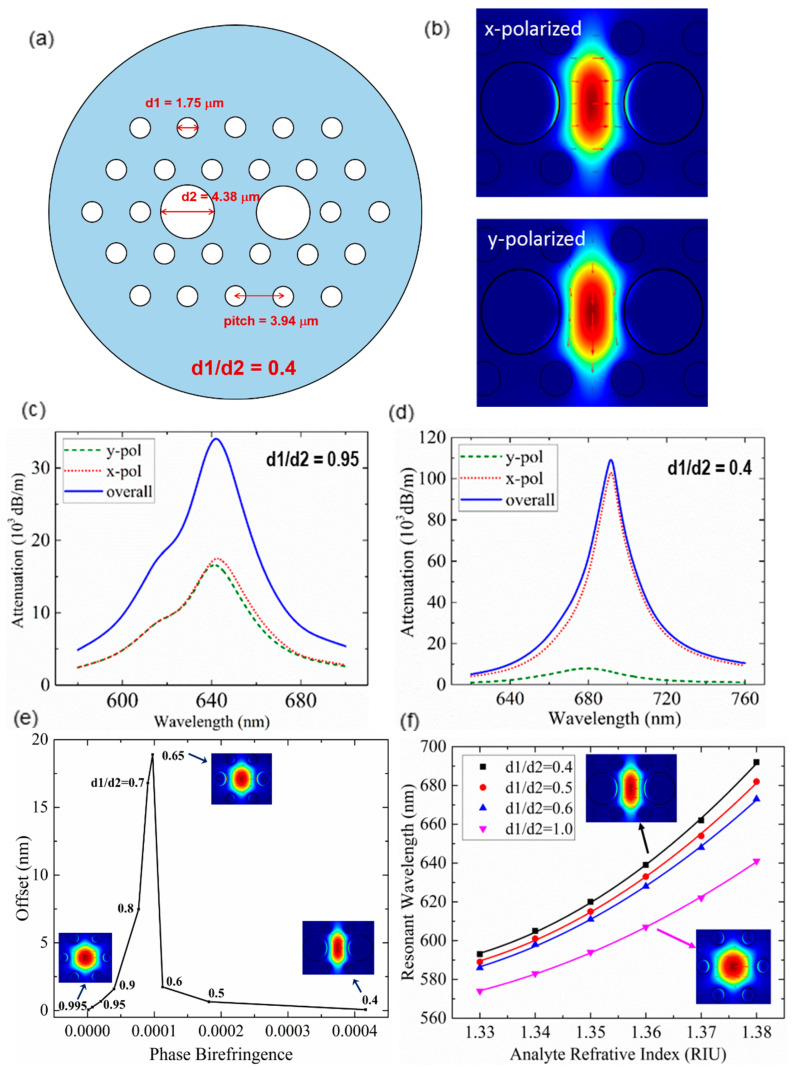
(**a**) The configuration of proposed highly birefringent microstructured optical fiber (MOF). (**b**) x-polarized and y-polarized core mode pattern of the SPR sensor (d1/d2 = 0.4) (Figure adapted with permission from reference [31]). (**c**) Attenuation spectra of highly birefringent MOF when d1/d2 = 0.95 and (**d**) d1/d2 = 0.4. (**e**) The variation of wavelength offset along with phase birefringence. (Inset) The x-polarized core mode pattern. (**f**) The SPR sensitivities when d1/d2 = 1.0, 0.6, 0.5 and 0.4 respectively. (Inset) The x-polarized core mode (Figure adapted with permission from reference [32]).

To address the issue of birefringence induced measurement instability, polarization-maintaining MOF-based SPR sensor with high birefringence could be a promising solution. A large birefringence can be realized in a near-panda MOF with the two central air holes of the photonic-crystal arranged cladding holes enlarged (Figure 3a). The material of the MOF is fused silica. The enlarged two central holes can facilitate easier thin noble metal film deposition and analyte infiltration. Strong surface plasmons can be excited by the x-polarized fundamental core mode with the thin gold film deposited on the inner walls of central holes. As discussed earlier, SPP can only be excited by the TM-polarized incident light (i.e., the polarization perpendicular to the metal film surface), and y-polarized core mode corresponds to a much weaker SPP compared with that of x-polarized mode (Figure 3b). This indicates the SPR sensing output is predominated by the plasmonic behaviors of x-polarized code mode. For a low-birefringent MOF, of which the diameter of central holes (d2) is comparable to that of other cladding holes (d1) (e.g., d1/d2 = 0.95), both x- and y-polarized mode can excite relatively strong SPP. As a result, the existence of unwanted polarization could induce an offset of overall resonant wavelength as high as 0.67 nm from that of the desired polarization, which means the SPR sensing accuracy is considerably compromised (Figure 3c). On the contrary, even though a highly-birefringent MOF consists of two modal polarizations corresponding to even larger resonant wavelength difference, the immensely suppressed SPP of unwanted polarization has a bare influence on the overall resonant wavelength and the sensing accuracy. For instance, the wavelength offset of the proposed highly birefringent near-panda MOF with d1/d2 = 0.4 is as small as 0.06 nm (Figure 3d).

Based on the finite element method (FEM) simulation of photonic-crystal arranged MOFs with different d1/d2 ratios, the relation between phase birefringence and sensing inaccuracy can be deduced. As shown in Figure 3e, the resonant wavelength offset could increase to be as large as 18.89 nm when the phase birefringence increases from ~4 × 10^−5^ to ~1 × 10^−4^. When the phase birefringence exceeds beyond a threshold (~1 × 10^−4)^, the wavelength offset effectively reduces and even tends toward 0 after 4 × 10^−4^ phase birefringence. The investigation indicates that small birefringence that commonly exists in MOF-based SPR sensors could induce non-negligible undesired resonant wavelength offset, which affects sensing accuracy. The proposed highly-birefringent MOF with intentionally introduced large phase birefringence ~4.2 × 10^−4^ can effectively suppress such impact of polarization crosstalk to be extremely small. In addition, more expanded central holes enhance the plasmon–matter interaction, thereby providing higher sensitivity. Figure 3f compares the sensitivities when d1/d2 = 0.4, 0.5, 0.6 and 1.0. It is clear that the sensitivity is improved when the central holes expand. At a high analyte refractive index range of 1.37–1.38, the proposed highly-birefringent MOF SPR sensor can achieve a sensitivity as high as 3000 nm/RIU. 

**Figure 4:**
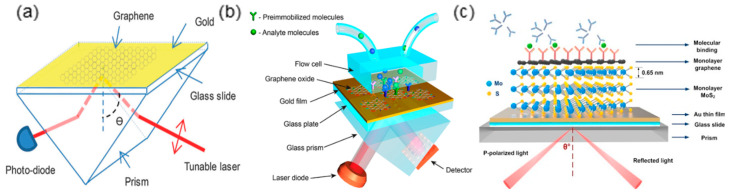
The prism based SPR configurations with hybrid plasmonic structures of (**a**) single-layer graphene/gold (Figure adapted with permission from reference [39]); (**b**) multilayer graphene/Py/gold (Figure adapted with permission from reference [42]); (**c**) graphene-MoS2/gold (Figure adapted with permission from reference [46]).

Besides optimizing the design of fiber structure, integrating functional nanomaterials with a fiber-optic platform can also effectively promote the light-matter interaction. In the past decade, 2D materials have drawn extensive attention in various research fields including the highly integrated sensors. The extremely large surface-to-volume ratio, in situ plasmonic properties tunability and near field confinement are the great advantages of 2D materials in sensing applications [33,34,35,36]. The plasmonics of most common 2D materials such as graphene and MX_2_ fall in MIR or terahertz regions, which are not compatible with the well-developed optical communication window even though they can achieve superior plasmonic sensing performance [37,38]. Therefore, numerous research efforts focus on enhancing the plasmon–matter interaction by applying 2D material/metal film hybrid structures to SPR configurations. For instance, the thin gold film in conventional Kretschmann configuration has been upgraded to graphene/gold [6,39,40,41], graphene oxide/gold [42,43,44,45], graphene-MoS_2_/gold [46], etc. hybrid film-like architectures (Figure 4). It is proven that the intensity of SPP on the gold film surface can be effectively strengthened by the seamlessly integrated graphene layer. When graphene and gold are in contact, the work function difference between the two materials (4.5 eV for graphene and 5.54 eV for gold) causes electrons to flow from graphene to gold to equilibrate the Fermi levels [47,48]. As a result, the electron density at the gold film surface increases as the graphene becomes p-type doped. Therefore, a stronger SPP so a higher sensitivity can be achieved.

**Figure 5:**
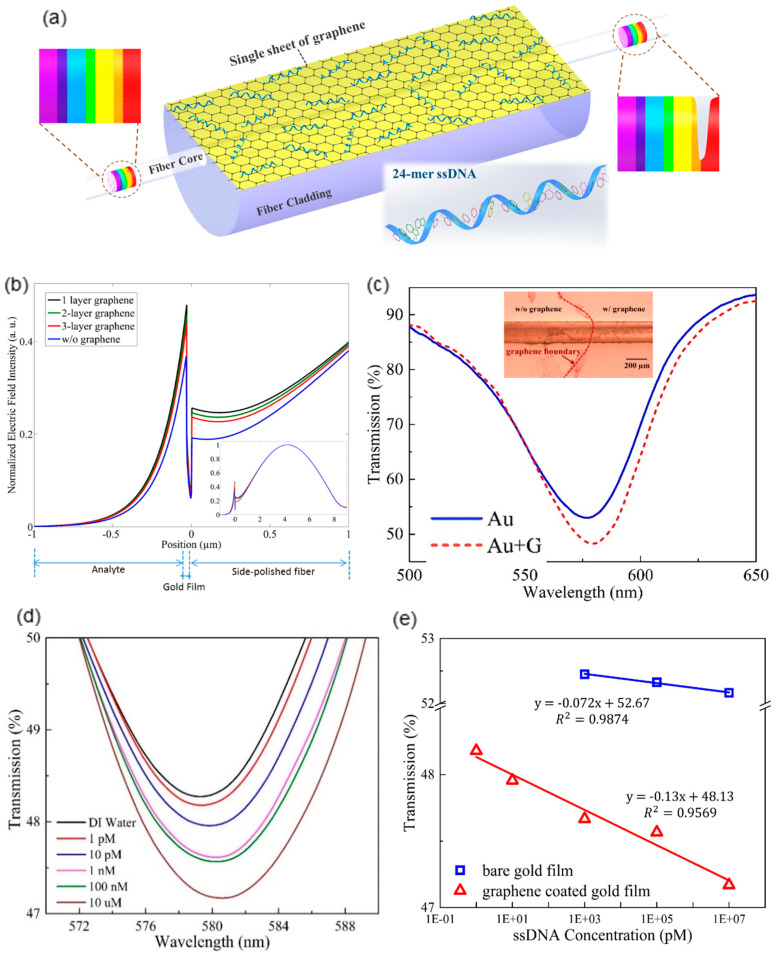
(**a**) The schematic illustration of proposed hybrid graphene-on-gold SPR sensor. The nucleobases of target ssDNA molecules can form stable π-stacking interaction with the honeycomb arrange carbon atoms of graphene. (**b**) The comparison of electric field intensities when the SPP is excited by bare thin gold film, single layer graphene/gold, 2-layer graphene/gold and 3-layer graphene/gold. (Inset) The electric field distribution over the entire fiber-optic graphene/gold hybrid structure. (**c**) The comparison of fiber transmission spectra with and without single graphene layer (Inset) The microscopic view of the single graphene layer transferred on the side-polished fiber. (**d**) The transmission spectra variation of proposed hybrid plasmonic sensor along with the increase of ssDNA concentration. (**e**) The comparison of sensitivities to ssDNA concentration of fiber-optic plasmonic sensors with and without graphene layer (Figure adapted with permission from reference [51]).

Even though the 2D material/metal film hybrid structure had been widely proposed on the prism-based SPR configuration, systematic analysis and experimental demonstration of integrating such hybrid plasmonic structure with flexible waveguides such as optical fibers were rare. As a proof of concept, a graphene-on-gold hybrid structure is proposed to be seamlessly integrated with a side-polished optical fiber, purposing to demonstrate that the 2D material/metal hybrid structures could achieve enhanced plasmonic biosensing performance on flexible waveguide platforms. As illustrated in Figure 5a, the exposed evanescent field of guided core mode interacts with the graphene-on-gold structure deposited at the surface of polished facet of optical fiber, leading to strong SPP-biomolecules interaction. Meanwhile, the single graphene layer functions as excellent surface functionalization of the thin gold film. Since the SPP at the gold film surface exponentially decays with the penetration depth, the thickness of surface functionalization is a crucial factor that affects sensitivity. The graphene layer, as thin as 0.34 nm, could hardly compromise the SPR sensitivity [49]. Moreover, the carbon atoms of graphene arranged in honeycomb format can easily form π-stacking interaction with the aromatic rings commonly existed in biomolecules [50]. Hence it facilitates effective adsorption of target biomolecules such as ssDNA, providing high sensitivity and low limit of detection (LOD).

Simulation can verify the SPP enhancement capability of the additional graphene sheet on conventional gold film coated side-polished fiber. The inset of Figure 5b plots the whole electrical field distribution of guided core mode in the fiber as well as the SPP on the side-polished facet. The magnified field distribution of the SPP at the gold/graphene surface is shown in Figure 5b. As expected, introducing single or multiple graphene layers can effectively enhance the SPP intensity on the thin gold film surface, which benefited from the electrons transfer, as explained above. Another interesting finding in the simulation is that bilayer or multi-layer graphene slightly compromises the SPP intensity compared with the single-layer graphene. This is due to the electrons’ energy loss induced by the increase of graphene layers [49]. Therefore, with the SPP intensity boosted by ~30.2%, a single graphene layer most enhances the plasmonic sensing behavior. The experimental results further verify that the graphene-on-gold hybrid structure can effectively improve the plasmonic sensing behavior. Figure 5c compares the resonant peaks of the conventional thin gold film coated side-polished fiber and the graphene-on-gold hybrid structure integrated side-polished fiber when both sensing configurations are immersed in deionized (DI) water. The inset of Figure 5c shows the microscopic view of the boundary of transferred graphene on the thin gold film coated side-polished fiber facet. The graphene-on-gold hybrid structure corresponds to a deeper resonant peak, indicating a stronger SPP intensity, which matches well with the simulation.

The biosensing capability of the proposed plasmonic hybrid SPR configuration can be validated by detecting ssDNA concentration. ssDNA quantization provides biomedical significance in gene expression, DNA sequencing and polymerase chain reaction (PCR) [52]. Figure 5d shows the magnified SPR peaks of the biosensing platform with the incrementing ssDNA concentration. This can be explained by Equation (1) that the surrounding refractive index of the plasmonic architecture is increased due to the efficient adsorption of ssDNA molecules on the graphene surface via π-stacking interaction. Also, the SPP evanescent field is scattered by the bonding of ssDNA molecules, which further induces transmission loss, thereby a deeper SPR peak. The LOD of the biosensor to ssDNA molecules is as small as 1 pM based on the distinguishable enhancement of the SPR peak (the red curve of Figure 5d). To experimentally verify that the biosensing performance is improved by the additional graphene layer, a conventional thin gold film based side-polished fiber-optic SPR sensor is prepared and applied to measure the same ssDNA solutions. The comparison of sensitivities corresponding to the two structures in Figure 5e can obviously indicate that the graphene-on-gold hybrid structure can effectively improve the sensitivity almost two-fold.

**Figure 6:**
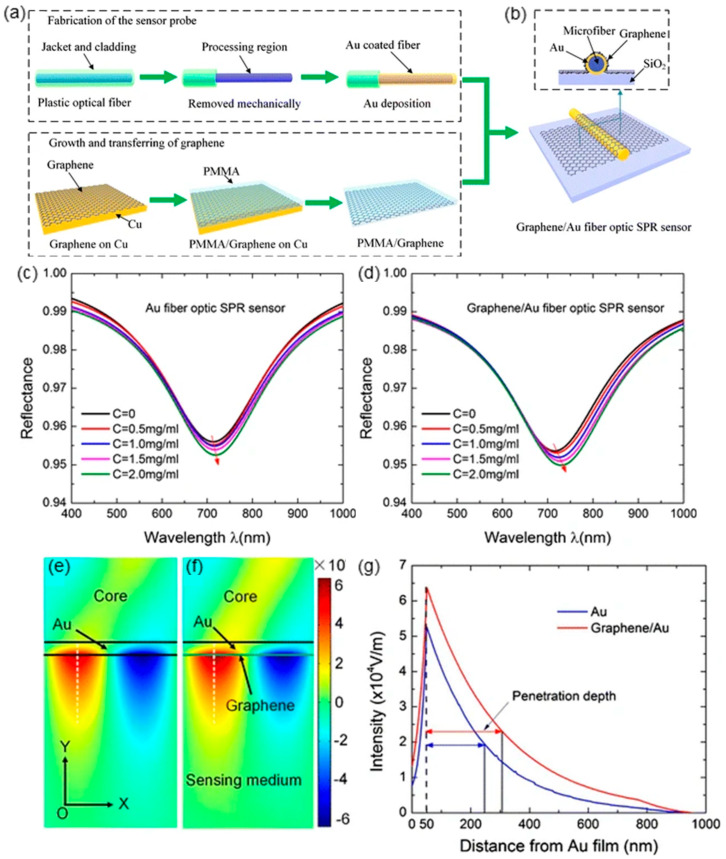
(**a**) Fabrication of Au coated fiber and preparation of graphene monolayer. (**b**) The schematic illustration of cross-section view of the proposed graphene/Au fiber-optic SPR sensor. (**c**) Reflection spectra of Au (**c**) and graphene/Au (**d**) fiber-optic SPR sensor with varying bovine serum albumin (BSA) concentration. (**e**) Finite element analysis (FEA) simulation of electric field distribution of fiber-optic SPR sensors with (**e**) and without (**f**) graphene. (**g**) Electric field decaying along Y-direction (Figure adapted with permission from reference [6]).

Wei et al. also compared the theoretically and experimentally performances of the fiber-optic SPR sensors with and without graphene in evaluating bovine serum albumin (BSA) concentration [6]. As shown in Figure 6a,b, the unclad portion of a plastic optical fiber is deposited with a gold film, and the graphene monolayer is transferred to the gold surface by PMMA. Figure 6d shows the variation of the reflection spectra of the graphene/Au fiber-optic SPR sensor with BSA concentration ranging from 0 to 2 mg/mL, which displays a 13.8-nm redshift compared with 6.1 nm for the Au fiber-optic SPR sensor (Figure 6c). After linearly fitting the resonant wavelengths and BSA concentration, the sensitivity of the graphene/Au hybrid sensor is 7.01 nm/(mg/mL), while the sensor without graphene is only 2.98 nm/(mg/mL). Additionally, regarding the full width half maximum (FWHM) of the two sensors, the graphene/Au hybrid sensor also possesses a more obvious variation tendency.

The finite element analysis (FEA) method based on COMSOL Multiphysics is established to clarify the improved sensing capability by graphene. The calculated electric field mode diagrams of fiber-optic SPR sensors with and without graphene are displayed in Figure 6e,f, respectively. On the sensing medium/Au interface, both sensors exhibit the similar confined electric field distributions, while the presence of graphene can strengthen the confined electric field with a maximum intensity of 6.4 × 10^4^ V/m. Furthermore, in Figure 6g, the electric field intensity of the two structures perpendicular to the sensing interface (white dashed line) is extracted and compared. As can be seen, both electric field intensity exponentially decays along with the distance from Au film, and graphene/Au hybrid structure reveals a more considerable penetration depth of 256 nm, thus improving the sensitivity to the surrounding medium.

**Figure 7:**
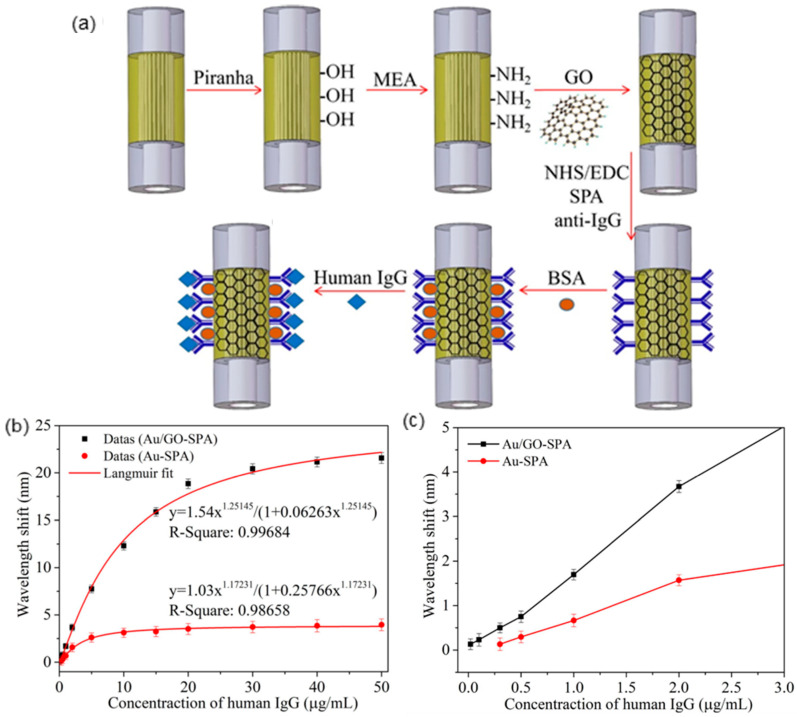
(**a**) Fabrication of graphene oxide (GO)-modified SPR immunosensor. (**b**) Fitting curve of wavelength shift versus human IgG concentration. (**c**) Local enlarged drawing (Figure adapted with permission from reference [44]).

Based on the same mechanism, Wang et al. developed an SPR immunosensor employing graphene oxide (GO)-modified photonic crystal fiber (PCF) for human IgG detection [44]. PCF is a type of MOF consisting of a honeycomb structure with air holes, infiltrating liquid crystals into these air holes enables PCF tunable optical characteristics [53,54,55,56,57,58,59,60]. As shown in Figure 7a, the PCF with five layers of air holes is spliced between two multimode fibers (MMFs). After being deposited with Au film, the fiber is cleaned with piranha and then modified with Mercapto ethylamine (MEA) to enrich amine (-NH_2_) groups for further reaction with epoxy groups on GO. Subsequently, the EDC/NHS system is used to activate the carboxyl of GO, and anti-IgG is directionally linked by staphylococcal protein A (SPA) orientation. Finally, BSA is introduced to block the free SPA surface, and the sensor is ready for human IgG sensing.

The immune reaction between anti-IgG and human IgG will cause wavelength redshift, as shown in Figure 7b, the wavelength shift and human IgG concentration can be fitted using the Langmuir equation. Compared with the Au-SPA sensor, Au/GO-SPA sensor exhibits a distinct redshift of 0.02 nm to 21.57 nm. After zooming in (Figure 7c), it can be observed that the LOD of Au/GO-SPA sensor (0.01 µg/mL) is 30 times lower than the Au-SPA sensor (0.3 µg/mL), which indicated GO significantly enhanced the immunosensor sensitivity.

**Figure 8:**
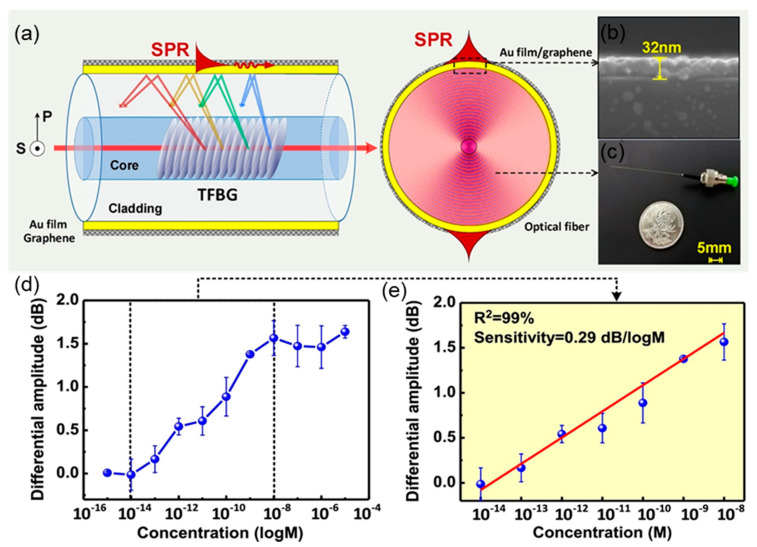
(**a**) The schematic illustration of proposed graphene/Au TFBG fiber-optic sensor (polarimetric sensing characteristic of TFBG and the energy distribution along fiber cross section). (**b**) Scanning electron microscope (SEM) image of graphene monolayer coated on Au surface. (**c**) photograph of the whole fiber-optic probe. (**d**) The differential amplitude output versus dopamine concentration. (**e**) The linear relationship between differential amplitude and dopamine concentration. (Figure adapted with permission from reference [41]).

Hu et al. also incorporate the graphene monolayer on a gold-coated TFBG (Figure 8a), the TBFG is further functionalized with ssDNAs by π–π stacking for dopamine detection. As shown in Figure 8d, there is an obvious differential amplitude increase when dopamine concentration raises from 10^−14^ M to 10^v13^ M, and a quite linear correlation (R^2^ = 99%) is observed over dopamine concentration from 10^−13^ M to 10^v8^ M. TFBG enables the optic-fiber sensor with high RI sensitivity, narrow cladding and innate insensitivity to temperature and optical power fluctuations, which is feasible for biomedical sensing.

Although the 2D material/metal hybrid structures facilitate remarkable light-matter interaction in plasmonic sensing, the intrinsic SPP of most common 2D materials (e.g., graphene and MX_2_) located at the MIR range is almost impossible for practical applications. Therefore, an alternative class of 2D plasmonic material, heavily doped ultrathin TMOs, have captured research attention in recent years aiming for manipulating the intrinsic plasmonics of 2D materials with exceptional field confinement and in situ plasmonic tunability in the frequently used visible and NIR optical window [61,62,63,64]. To realize SPP in visible or NIR frequencies, sufficient free carrier concentration must be achieved in 2D materials. The unique character of outer-d valence electrons enables TMOs to achieve sufficient free carrier doping via ionic intercalation. Taking the most representative TMOs, molybdenum trioxide (MoO_3_) and tungsten oxide (WO_3_), as examples, free electrons can be abundantly doped by introducing oxygen vacancies in the TMO lattice [65,66]. Therefore, the plasmonic behavior of 2D TMOs can be easily tuned by manipulating the oxygen vacancies. So far, the tunable plasmonics of heavily doped MoO_3_ nanoflakes in visible or NIR region has been most widely studied, yet the exploration on integrating such emerging 2D materials with optical devices especially the highly-integrated waveguide based sensing devices is very limited.

**Figure 9:**
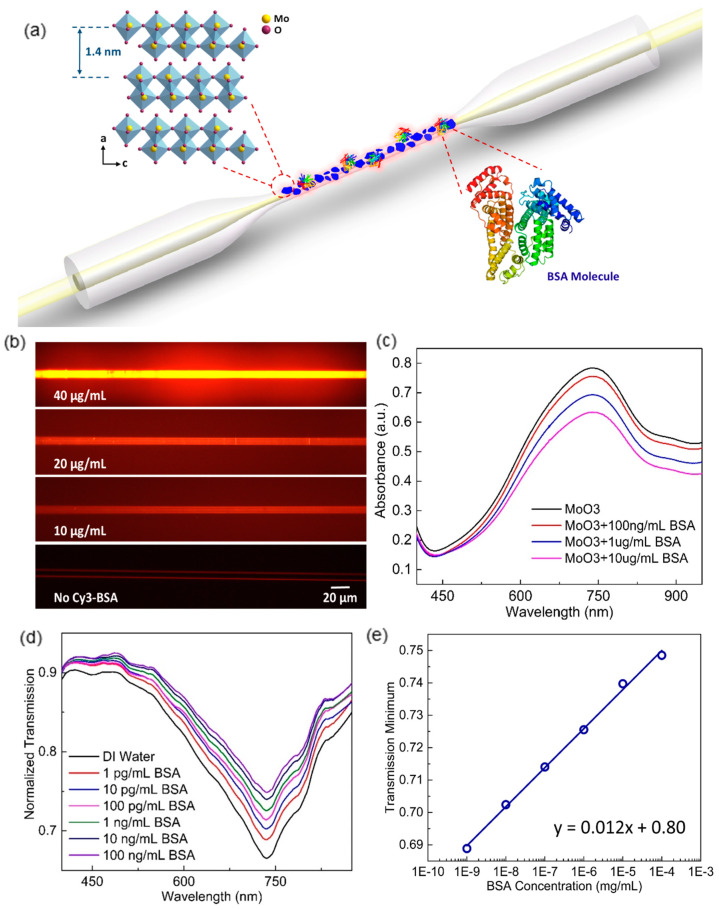
(**a**) The schematic illustration of heavily-doped MoO_3−x_ nanoflakes based hybrid fiber-optic plasmonic biosensor. (Inset 1) The crystal structure of α-MoO_3_ lattice. (Inset 2) Molecular structure of BSA. (**b**) The fluorescent microscopic view of MoO_3−x_ nanoflakes functionalized microfibers coated with different concentrations of dye labelled BSA molecules. (**c**) The absorption spectra of MoO_3−x_ nanoflakes solutions mixed with different BSA concentrations. (**d**) Transmission spectrum variation of proposed hybrid plasmonic biosensor along with increasing BSA concentration. (**e**) The linear increase of transmission minimum against log-scale BSA concentration (Figure adapted with permission fromreference [68]).

Driven by the purpose of investigating the potential of 2D TMOs on highly-integrated plasmonic devices, a biosensor based on a microfiber functionalized with α-MoO_3_ nanoflakes is developed and validated by BSA molecules detection. As shown in Figure 9a, few-layer α-MoO_3_ nanoflakes are synthesized by the liquid-phase exfoliation method [67] and then heavily doped with free electrons via an H^+^ intercalation process [68]. After doping, a sub-stoichiometric α-MoO_3−x_ nanoflakes solution with strong SPP at the NIR region is formed. Pristine MoO_3_ only introduces absorption at ultraviolet (UV) wavelengths, which is due to the large bandgap of 3.2 eV [69]. After electrons are increasingly doped, a distinct absorption peak appears and enhances at 700–800 nm range, in the meantime, undergoes a blueshift. This phenomenon can be explained by Drude model that the plasma frequency is inversely correlated to electron density [70,71].

The MoO_3−x_ nanoflakes can be stably immobilized on the microfiber surface via electrostatic interaction. Since MoO_3−x_ is positively charged, the microfiber surface functionalized with evenly distributed negative charges (e.g., self-assembled poly(allylamine) (PAA)/poly(styrene sulfonate) (PSS) bilayer) applies strong attraction to the nanoflakes. Similarly, the immobilized positively charged MoO_3−x_ nanoflakes on the microfiber surface can effectively attract negatively charged target molecules, such as BSA [67]. Dye-labeled BSA molecules are adopted to verify the effectiveness of electrostatic interaction-based target molecule adsorption as well as fiber surface functionalization. Figure 9b shows the fluorescent microscope views of four MoO_3−x_ nanoflake-deposited microfibers after immersing in different concentrations of dye-labeled BSA solutions. It is evident that the fiber brightens as the BSA concentration increases. Also, the even brightness on the fiber surface implies the uniformity of adsorbed BSA molecules so as the MoO_3−x_ nanoflakes.

The binding of negatively charged BSA molecules on the MoO_3−x_ nanoflakes surface impacts the plasmonic behavior. When MoO_3−x_ nanoflakes suspensions mix with different concentrations of BSA solution, the absorption peak of MoO_3−x_ weakens as the BSA concentration increases (Figure 9c). This is due to the free electrons at the MoO_3−x_ surface being repelled by the negatively charged BSA molecules, resulting in the reduced free electron density involved in the plasmonic resonance [61,64,72]. Therefore, a fiber-optic sensor based on MoO_3−x_ nanoflakes shows a unique characteristic that the resonance peak on the fiber transmission spectrum gradually shallows along with the increasing concentration of target BSA (Figure 9d). Profited from the full utilization of a high aspect ratio of 2D MoO_3−x_, a LOD of BSA as low as 1 pg/mL is achieved. Moreover, the transmission minimum of the plasmonic resonance peak provides a linear response to the log-scale BSA concentration (Figure 9e).

With the vigorous development of material science, there is abundant research to introduce diverse materials into fiber-optic SPR sensors [73,74,75,76,77]. For instance, Santos et al. propose a refractive index sensor by combining Al_2_O_3_-Ag metamaterial film with D-type PCF fiber, in which the sensor performance can be adjusted by the thickness and component of metamaterial [78]. Semwal et al. wrapped Ag-coated optical fiber with the enzyme (ADH) and coenzyme (NAD)–containing hydrogel to establish an ethanol sensor. These will surely boost the advancement of fiber-optic fiber sensors [79].

## 3. Fiber-Optic Localized Surface Plasmon Resonance Sensors

**Figure 10:**
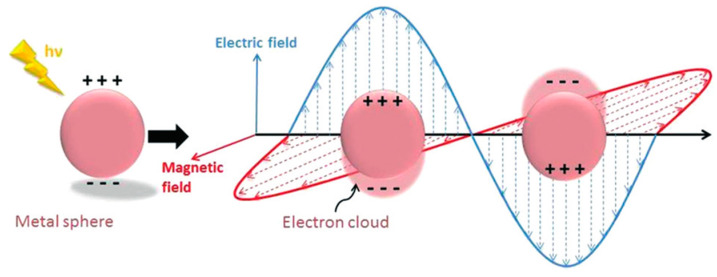
The schematic diagram of localized surface plasmon resonance (Figure adapted with permission from Ref. [81]).

By contrast with the propagating SPP at a thin metal film surface, the resonant electron oscillation induced by light interacting with a metallic nanoparticle is non-propagating due to the particle size restriction. Therefore, it is called localized SPR (LSPR). LSPR can be excited when the oscillation frequency of nanoparticle electron cloud matches with the frequency of incident light (Figure 10) [80,81]. A proper model for understanding how incident light is scattered and absorbed by a nanoparticle with a diameter much smaller than the wavelength is the Mie theory. The Mie theory constructs a model to deduce the extinction cross-section of nanoparticle based on the assumption that the nanoparticle is a homogeneous conducting sphere:

(5) $$\sigma_{ext}=9\left(\frac{\omega }{c}\right){\left(\varepsilon_{dielectric}\right)}^{\frac{3}{2}V}\frac{\varepsilon_{metal}^{\prime \prime }}{{\left(\varepsilon_{metal}^{\prime }+2\varepsilon_{dielectric}\right)}^{2}+{\left(\varepsilon_{metal}^{\prime \prime }\right)}^{2}}$$

where *V* is the volume of nanoparticle, and *ε’_metal_* and *ε”_metal_* are the real and the imaginary parts of the metal-dielectric function, respectively, in the Drude model [82]:

(6) $$\varepsilon_{metal}^{\prime }=1-\frac{\omega_{p}^{2}}{\left(\omega^{2}+\gamma^{2}\right)}$$

(7) $$\varepsilon_{metal}^{\prime \prime }=\frac{\omega_{p}^{2}\gamma }{(\omega^{2}+\gamma^{2})\omega }$$

where *γ* is the damping of electron oscillation and *ω_p_* is the bulk plasma frequency. More detailed definitions of *γ* and *ω_p_* can be found in [83]. Since LSPR operation frequencies are generally within the visible and NIR optical windows where *γ*≪*ω_p_*, Equation (7) can be simplified as:

(8) $$\varepsilon_{metal}^{\prime }=1-\frac{\omega_{p}}{\omega^{2}}$$

Based on Equation (5), the resonance is satisfied (i.e., the extinction cross-section is maximum) when *ε’_metal_* = –2*ε_dielectric_*. The LSPR resonant frequency is thereby expressed as:

(9) $$\omega_{LSPR}=\frac{\omega_{p}}{\sqrt{2\varepsilon_{dielectric}+1}}$$

Furthermore, for dielectric medium, *ε_dielectric_* = *n^2^_dielectric_*. Therefore, the refractive index of the ambient dielectric medium of the nanoparticle impacts the LSPR resonant wavelength:

(10) $$\lambda_{LSPR}=\lambda_{p}\sqrt{2n_{dielectric}^{2}+1}$$

**Figure 11:**
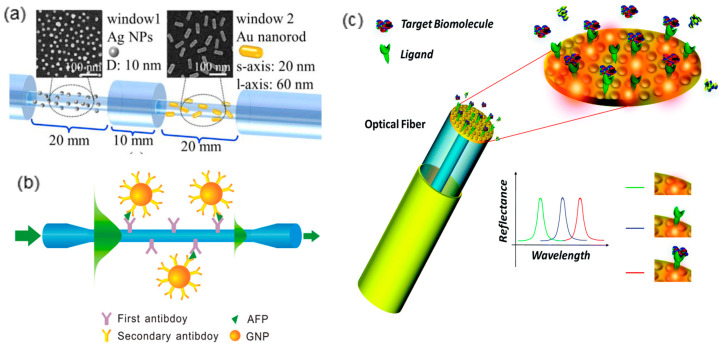
The LSPR devices based on various optical fiber structures such as (**a**) cascaded unclad fiber (Figure adapted with permission from reference [97]); (**b**) microfiber (Figure adapted with permission from reference [98]); (**c**) optical fiber endface (Figure adapted with permission from reference [99]).

Similar to SPR-based fiber-optic sensing platforms, LSPR-based fiber-optic devices have also captured intensive research attention [84,85,86,87,88,89]. Various fiber structures such as microfiber, cascaded unclad fiber, fiber endface, etc. have been integrated with silver or gold nanoparticles and shown promising plasmonic sensing performance (Figure 11). To achieve efficient selectivity to analyte molecules, it is necessary to apply surface functionalization on metallic nanoparticles. Taking the most widely employed gold nanoparticle as an example, many effective functionalization strategies have been proven, such as biomolecule coating [90,91,92], ligand substitution [93], polymer deposition [94,95], etc. However, when sensitivity is the crucial factor of plasmonic sensors, it is critical to keep the surface functionalization as thin as possible. Due to the evanescent nature of surface plasmon, thick surface functionalization considerably compromises the plasmon–matter interaction. For instance, a study has compared the LODs and sensitivities of two functionalization strategies with thicknesses of 4.24 nm and 0.96 nm respectively and shown that surface functionalization thinner than 1 nm significantly improves the sensing performance [96]. In such cases, macrocyclic supramolecules have shown the potential to meet the challenges of achieving both sub-nanometer functionalization thickness and target molecules recognition.

**Figure 12:**
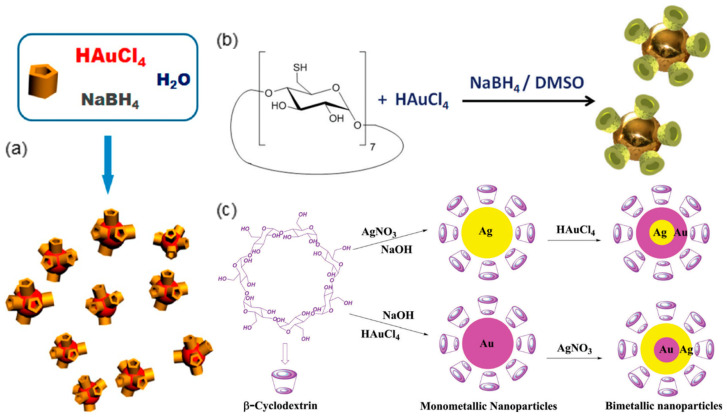
(**a**) The synthesis formulas of (**a**) carboxylatopillar[5]arene capped gold nanoparticles (AuNPs) ((Figure adapted with permission from reference [105]). (**b**) Cyclodextrin (CD)-capped AuNPs (Figure adapted with permission from Ref. [106]). (**c**) CD capped silver nanoparticles, AuNPs and Ag_core_-Au_shell_/Au_core_-Ag_shell_ bimetallic nanoparticles (Figure adapted with permission from reference [107]).

Benefiting from their macrocyclic cavities, macrocyclic supramolecules like cyclodextrins (CDs), cucurbiturils, pillararenes, calixarenes, etc. show excellent molecular recognition capability by the host-guest interaction. The host–guest interaction is noncovalent interaction between macrocyclic supramolecules and corresponding guest molecules to form inclusion complexations [100]. Encouragingly, it is proven that host–guest interaction is a more effective target molecule recognition and adsorption mechanism compared with the conventional biomolecule-ligand binding [101]. Another advantage of macrocyclic supramolecules being surface functionalization of metallic nanoparticles is the heights of their macrocyclic cavities being normally less than 1 nm [102,103,104], which facilitates sensitive molecular detection as discussed above. In addition, the macrocyclic supramolecules also eliminate the cytotoxicity of nanoparticles that is often favored in bio-medical sensing applications. In recent years, studies have been carried out to achieve functionalizing metallic nanoparticles with macrocyclic supramolecules during the synthesis of nanoparticles instead of through post-processing surface modification. In most of these attempts (Figure 12), however, harsh reducing reagents such as thiols, NaBH_4_, NaOH have to be introduced, which violates the purpose of achieving biocompatibility in many LSPR biosensing applications [105,106,107]. Therefore, inspired by the method proposed by Zhao et al. [108], where CDs act as both reducing and capping agent for AuNPs synthesis, a microfiber based LSPR biosensor is developed to comprehensively investigate the plasmonic sensing potential of one-step synthesized macrocyclic supramolecules decorated metallic nanoparticles. 

**Figure 13:**
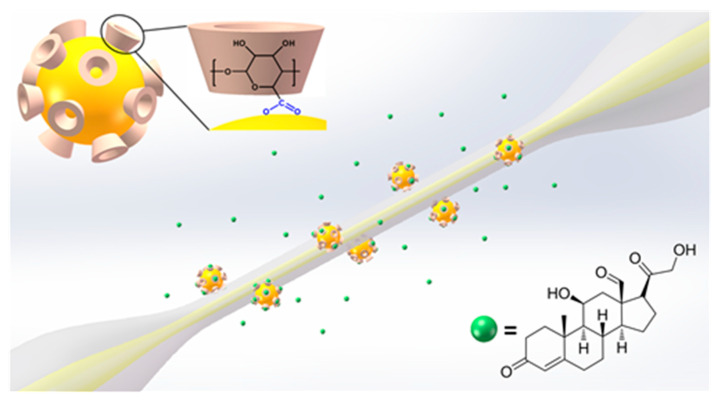
The schematic illustration of the β-CD-capped AuNPs based fiber-optic biosensor. (Inset1): The conjunction between β-CD molecule and AuNP surface. (Inset2): The molecular structure of cholesterol (Figure adapted with permission from reference [109]).

Figure 13 illustrates the configuration of LSPR biosensor based on a microfiber integrated with one-step synthesized β-CD-capped AuNPs [109]. Functioning as both the reducing and capping agent, β-CD facilitates the AuNPs formation and forms biocompatible functionalization layer via the conjunction of carboxyl groups and gold surface. The evanescent field of guide mode in microfiber leaks out at the tapered portion and interacts with immobilized AuNPs at the fiber surface, leading to a strong LSPR peak on the transmission spectrum. Cholesterol, the guest molecule of β-CD [108,110], is employed to validate the biosensing performance of the proposed LSPR device. The sterol groups of cholesterol molecules can tightly fit into the β-CD macrocyclic cavity, meanwhile forming stable host–guest interaction through the hydrophobic associations [111,112].

The as-synthesized β-CD-capped AuNPs show good uniformity in particle size with diameters range from ~18 nm to ~21 nm. The dynamic light scattering (DLS) characterization of the AuNPs further verifies the observation [109]. The resonance band of the absorption peak of β-CD-capped AuNPs solution with linewidth as narrow as 47 nm also indicates the monodispersity of the particles, which is comparable with that of conventionally synthesized AuNPs. Proton nuclear magnetic resonance (^1^H NMR) and Fourier transform infrared (FTIR) spectra are performed to further illustrate that the hydroxyl groups in β-CDs mainly contribute to reducing Au^3+^ ions to Au^0^ atoms [113].

**Figure 14:**
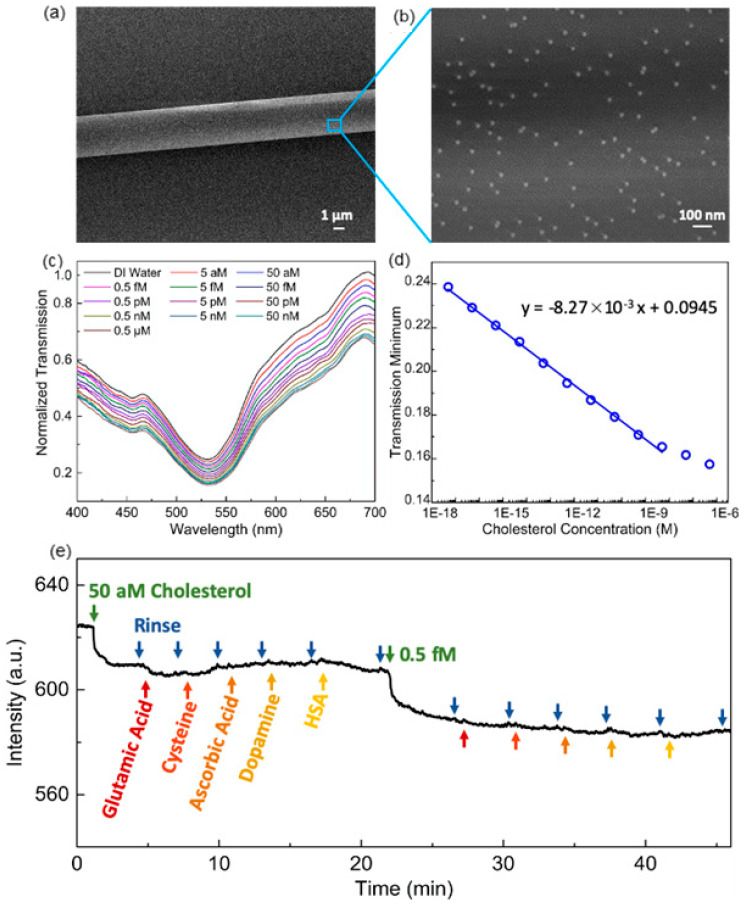
(**a**) The SEM image of 4-µm-diameter microfiber. (**b**) The distribution of β-CD-capped AuNPs on the microfiber surface. (**c**) Transmission spectrum variation of microfiber based hybrid plasmonic biosensor along with increasing cholesterol concentration. (**d**) The linear decrease of transmission minimum against log-scale cholesterol concentration. (**e**) The real-time average transmission intensity within 530–535 nm of microfiber when the interfering substances are introduced during cholesterol detection (Figure adapted with permission from reference [109]).

The as-synthesized AuNPs are negatively charged. Hence it can be stably immobilized on positively charged microfiber surface (e.g., functionalize the fiber surface with homogeneous PAA layer) via electrostatic attraction. As shown in Figure 14a,b the prepared microfiber is 4 μm in diameter and decorated with evenly distributed AuNPs. The attached AuNPs induce a deep resonance band centered at 530.7 nm on the microfiber transmission spectrum. When the fiber-optic sensing device is sequentially immersed in cholesterol solutions with concentrations ranging from 5 aM to 0.5 μM, the LSPR resonance band gradually deepens along with the increasing cholesterol concentration meanwhile the resonant wavelength shifts from 530.7 nm to 531.4 nm (Figure 14c). Such an ultra-low LOD of 5 aM is profited from the highly efficient host–guest interaction between β-CD and cholesterol. The transmission minimum of the resonance band can be taken as the sensing parameter and provides a linear response to the log-scale cholesterol concentration (Figure 14d).

The selectivity of the proposed biosensor to cholesterol is validated by an interference study, where common interfering substances in human serum such as glutamic acid, cysteine, ascorbic acid, dopamine and human serum albumin (HSA) are introduced. Figure 14e shows the real-time average transmission intensity within 530–535 nm of microfiber when the interfering substances are introduced during the detection of cholesterol. It is clear that the β-CD-capped AuNPs based fiber-optic sensor only responses to cholesterol molecules but not interfered by other substances. To further validate the cholesterol recognition capability of the proposed sensor, recovery experiments are also carried out to evaluate the accuracy of detecting real human serum samples diluted by a factor of 1014 and spiked with different cholesterol concentrations. As summarized in Table 1, the measurement of cholesterol concentration in the unspiked human serum sample is 4.23 mM. The measurement of the same sample using commercial blood cholesterol monitor is 4.35 mM, which indicates the proposed fiber-optic biosensor is reliable. In addition, the recoveries of the spiked samples are 105.2–112.2%, which is also within a satisfactory range, further verifies the accuracy of the proposed sensor. Therefore, it indicates the tremendous plasmonic sensing potential of highly integrated fiber-optic sensors based on the macrocyclic supramolecules modified metallic nanoparticles.

**Figure 15:**
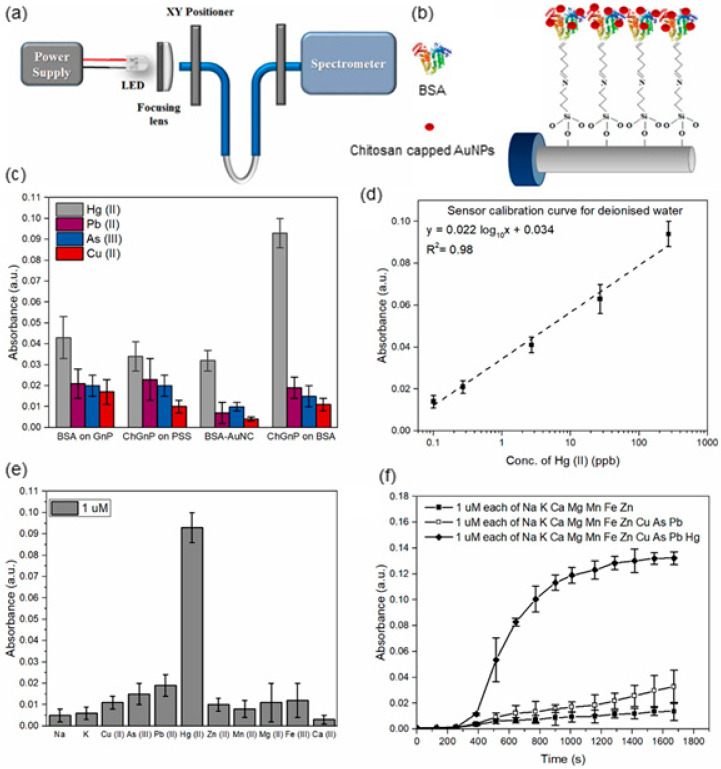
(**a**) The schematic illustration of the detection system. (**b**) Functionalization of chitosan-capped AuNPs on U-bent fiber. (**c**) Selection of optimal receptor for Hg(II) detection. (**d**) The linear increase of absorbance against Hg(II) concentration. (**e**) Absorbance at 520 nm for 1 µM individual metal ions detection. (**f**) Absorbance increasement against time for 1 µM metal ions mixture detection (Figure adapted with permission from reference [115]).

Another polysaccharide, chitosan, has also been used for AuNPs synthesis as a reducing and stabilizing agent [114]. Sadani et al. immobilize the synthesized chitosan-capped AuNPs (ChGnP) with a diameter of 20 nm on U-bent fiber for mercury (Hg(II)) detection [115]. The U-bent fiber is firstly incubating in (3-Aminopropyl)triethoxysilane (APTES) solution to enrich amine on the fiber surface, with glutaraldehyde crosslinking followed. Thereafter, BSA is linked to glutaraldehyde for further AuNPs’ immobilization (Figure 15a,b)

The sensitivity and selectivity of four sensors: BSA attaching to citrate capped AuNPs (BSA on GnP), polyanionic poly(sodium 4-styrenesulfonate) (PSS) immobilized ChGnP (ChGnP on PSS), fluorescent BSA-Au nanoclusters (BSA-AuNC) and BSA immobilized ChGnP (ChGnP on BSA) are compared. As shown in Figure 15c, compared to ChGnP on the BSA system, the first three show more deficient absorbance at the same Hg(II) concentration, and only the BSA-AuNC exists insignificant selectivity. The proposed ChGnP on the BSA LSPR sensor shows a linear calibration curve from Hg(II) concentration 0.1 ppb to 540 ppb (Figure 15d). 1 µM of different metal ions are dissolved in DI water separately, the absorbance for Hg(II) is greater than 0.9 a.u. while all other control ions are less than 0.2 a.u. (Figure 15e). Also, as shown in Figure 15f, when 1 µM of diverse metal ions mixtures with and without Hg(II) are detected, the change of absorbance at 520 nm over time shows that only mixture with the presence of Hg (II) reveals significant enhancement, further proving the excellent selectivity. The chemisorbed of Hg(II) on lone pair electrons of N, O and S atoms in chitosan and BSA, the hydrophobic interaction and the Van-der-Waals interaction with thiol groups in BSA are hypothesized the dominant factors of sensitivity and selectivity towards Hg(II).

**Figure 16:**
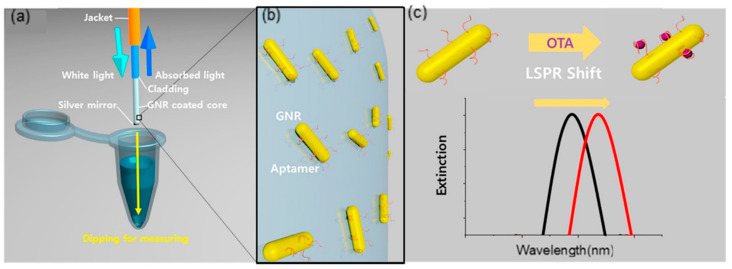
(**a**) The schematic illustration of the fiber-optic LSPR aptasensor. (**b**) Localized magnification of the fiber surface with GNPs immbolized on. (**c**) LSPR shift after ochratoxin A (OTA) recognization (Figure adapted with permission from reference [116]).

Lee et al. fabricate a fiber-optic LSPR sensor for the detection of ochratoxin A (OTA) utilizing aptamer-modified gold nanorods (GNRs) [116]. The GNRs are immobilized on the optical fiber by Au−S interaction, after being dipped into OTA solution, the LSPR spectrum is monitored exploiting the light reflection of a silver mirror at the end of the fiber. The aptamer’s specific recognition of OTA induces an LSPR peak shift (Figure 16c), and OTA can be specifically and quantitatively detected with a LOD of 12.0 pM and excellent linear response. This fiber-optic LSPR sensor possesses superior simplicity, which only demands to dip into OTA solution. The methods and performances of the hybrid fiber-optic sensors referred to are summarized is Table 2.

## 4. Conclusions

As discussed in this review, the proper design of MOFs with high birefringence provides wide possibilities in highly integrated microfluidic sensing devices with improved measurement accuracy and stability. Profiting from 2D material-based hybrid plasmonic structures, fiber-optic plasmonic sensors can deliver more promising sensing capability. The exceptional surface to volume ratio, near-field confinement and in situ plasmonic properties tunability of 2D materials facilitate the further enhancement of plasmon-matter interaction so as to enhance the sensitivity and LOD. In addition, the development of supramolecular chemistry brings new solutions in LSPR nanoparticles surface functionalization, leading to excellent target molecule selectivity via host–guest interaction. Given the numerous possibilities in optical design and hybrid plasmonic architectures construction, fiber-optic hybrid plasmonic sensors possess vast potential in various sensing scenarios with distinct advantages of high sensitivity, flexibility, miniaturization and high degree of integration. To further integrate the advances of specialty fibers and various nanomaterials, one major direction in the future is to achieve advanced functional fiber-based sensing by integrating multi-functional materials inside a single fiber or large-scale fabrics [117,118,119,120,121,122,123,124,125,126,127,128,129,130,131,132,133,134,135].

## Figures and Tables

**Table 1:** Recovery results of detecting cholesterol in human serum samples.

Sample	Added (aM)	Found * (aM)	Recovery (%)
Human serum (male)	0.0	42.3 ± 2.8	-
	50.0	94.9 ± 6.7	105.2
	100.0	154.5 ± 16.5	112.2

* The values are mean of 4 independent experiments ± standard deviation (Reprinted with permission from reference [110]).

**Table 2:** Summary of hybrid fiber-optic sensors in this review.

Material	Mode	Analyst	LOD	Remark	Ref
Graphene/Au	SPR	ssDNA	1 pM	π-Stacking with graphene	[51]
Graphene/Au	SPR	BSA	NA	Significance of graphene	[6]
GO/Au	SPR	Human IgG	0.01 µg/mL	Anti-IgG/IgG interaction	[44]
Graphene/Au	SPR	Dopamine	10^−13^ M	π-Stacking with ss-DNA	[41]
MoO_3−x_ nanoflake	SPR	BSA	1 pg/mL	Electrostatic interaction	[68]
β-CD/AuNPs	LSPR	Cholesterol	5 aM	Host-guest interaction	[109]
Chitosan/AuNPs	LSPR	Hg(II)	0.1 ppb	Chemisorbed	[115]
Aptamer/GNRs	LSPR	OTA	12.0 pM	Aptamer’s specific recognition	[116]
